# Carryover effects of long-distance avian migration are weaker than effects of breeding environment in a partially migratory bird

**DOI:** 10.1038/s41598-020-80341-x

**Published:** 2021-01-13

**Authors:** Claire Buchan, James J. Gilroy, Inês Catry, Javier Bustamante, Alina D. Marca, Philip W. Atkinson, Juan Miguel González, Aldina M. A. Franco

**Affiliations:** 1 School of Environmental Sciences, University of East Anglia, Norwich, Norfolk UK; 2 CIBIO/InBIO, Centro de Investigação em Biodiversidade e Recursos Genéticos, Laboratório Associado, Universidade do Porto, Campus Agrário de Vairão, 4485-661 Vairão, Portugal; 3 CIBIO/InBIO, Centro de Investigação em Biodiversidade e Recursos Genéticos, Laboratório Associado, Instituto Superior de Agronomia, Universidade de Lisboa, Tapada da Ajuda, 1349-017 Lisbon, Portugal; 4 Department of Wetland Ecology, Remote Sensing and GIS Lab (LAST-EBD), Estación Biológica de Doñana (CSIC), C/ Américo Vespucio 26, 41092 Sevilla, Spain; 5 British Trust for Ornithology, The Nunnery, Thetford, Norfolk UK; 6 Tumbabuey Grupo de Anillamiento, Cádiz, Spain

**Keywords:** Ecology, Animal migration, Behavioural ecology

## Abstract

Migration may expose individuals to a wide range of increasing anthropogenic threats. In addition to direct mortality effects, this exposure may influence post-migratory reproductive fitness. Partial migration—where a population comprises migrants and residents—represents a powerful opportunity to explore carryover effects of migration. Studies of partial migration in birds typically examine short-distance systems; here we studied an unusual system where residents breed in mixed colonies alongside long-distance trans-Saharan migrants (lesser kestrels (*Falco naumanni*) in Spain). Combining geolocator data, stable isotope analysis and resighting data, we examined the effects of this stark difference in migratory strategy on body condition, breeding phenology and breeding success. We monitored four colonies in two regions of southern Spain for five consecutive years (2014–2018), yielding 1962 captures, determining migratory strategy for 141 adult bird-years. Despite a 3000-km difference in distance travelled, we find no effect of strategy on breeding parameters. We find weak evidence for a short-term negative carryover effect of migration on body condition, but this was only apparent in the breeding region with lower primary productivity. Our results indicate that carryover effects of even highly divergent migratory strategies may be minimal relative to effects of conditions experienced on breeding grounds.

## Introduction

Migration represents a significant seasonal undertaking with potential for strong carryover effects^1,2^. Environmental conditions during spring migration can influence arrival time and body condition at the breeding grounds, with consequences for subsequent breeding success. Poor body condition following migration can lead to reduced resource investment in reproduction^3^, or later arrival on breeding grounds due to less time-efficient migration^4^. Breeding success is generally lower for later breeding attempts^5^, via (likely interacting) mechanisms relating to seasonal deterioration in conditions, correlates of individual quality, and lost opportunity for additional breeding attempts^6^. Climatic conditions experienced in the winter^7^ and while on migration^8^ can influence breeding phenology, while stresses experienced on spring staging grounds has been linked to subsequent lower breeding success^9^.

Given the potential for migratory carryover effects to be cumulative, between-individual differences in the magnitude of carryover effects might be expected in populations where individuals differ markedly in migratory behaviour. Migratory routes, distances and timings can vary significantly within populations^10^, sometimes leading to significant variation in survival^11^. Such differences may also apply to non-lethal carryover effects; larger migratory distances have been linked to later arrival on breeding grounds^12^ and lower breeding success^13^. Large differences in the magnitude of migratory movement within a population may therefore be expected to precipitate significant differences in subsequent fitness.

Partial migration, where migrant and non-migrant individuals exist within the same population^14,15^, provides a powerful natural experiment to explore these carryover effects, by comparing fitness parameters of migrants and residents^16^. Although parity in fitness under both strategies is necessary for the evolutionary maintenance of partial migration^14^ (especially if migratory strategy is heritable^17^), it is hypothesised that this balance is maintained through trade-offs where migratory costs that reduce fitness in one parameter (survival or breeding success) are compensated by higher fitness in another^14,18^. For instance, residents may suffer higher energetic costs of enduring colder climates and lower food availability, but enjoy higher reproductive success due to early access to breeding resources^19,20^. We may therefore expect to see differences in carryover effects between migrants and residents across different fitness components.

Anthropogenic change may also be influencing fitness differences within long-distance partially migratory populations. Migratory individuals can face greater exposure to threats (e.g. land-use change, extreme climatic events, novel infrastructure) than residents, which may interact and accumulate along migratory routes^21,22^. Simultaneously, climate warming and accompanying decreases in seasonality—at least in the northern hemisphere^23^—may favour residency^24^. Increasingly clement wintering conditions may also create phenological mismatches that disproportionately affect longer-distance migrants^25^. If anthropogenic change leads to a breakdown in the parity of fitness between migrants and residents it could lead to rapid changes in migratory behaviour, potentially ultimately leading to the disappearance of migration^26^. In light of the potential for cumulative carryover effects to manifest in the subsequent breeding season, we might expect long-distance migrants in partially migratory populations to be in worse condition than residents, breeding later and with lower reproductive fitness.

Most studies of within-population partial migration in birds are in short-distance systems^16^. Here we examine carryover effects in an unusual example of long-distance partial migration (lesser kestrels, *Falco naumanni*), where non-migratory individuals are fully resident in the Spanish breeding grounds throughout the year, while migrants undertake a c. 3000-km trans-Saharan migration, such that individuals may be exposed to very different costs between the two strategies. We combine geolocator tracking, ring-resighting and stable isotope analysis to determine migratory strategies of individuals, and examine effects on body condition and breeding parameters. We hypothesise that stark differences in migratory strategy will lead to measurable differences in each of these parameters, reflecting the contrasting winter conditions experienced. We additionally compare the strengths of these differences relative to colony-specific differences in breeding environment.

## Results

### Determining strategies

We were able to determine the migratory strategy (migrant or resident) of 116 individuals across 151 bird-years (90 residents, 61 migrants—see Supplementary Table S1). Of these, 107 individuals were adults, yielding 141 bird-years (81 residents, 60 migrants), which we carried forward to subsequent analyses (Supplementary Fig. S1). Contrary to previous findings^27^, we found no evidence for residents being disproportionately male, with approximately balanced sex ratios in both residents (44 males, 37 females) and migrants (27 males, 33 females). Of 25 adult individuals (59 bird-years) for which we determined migratory strategy in multiple years (17 individuals for two years, seven for three years and one for four years), 21 maintained a consistent migratory strategy, three individuals switched from migrants to residents, and one individual switched from being a resident to being a migrant.

### Adult body condition

**Figure 1:**
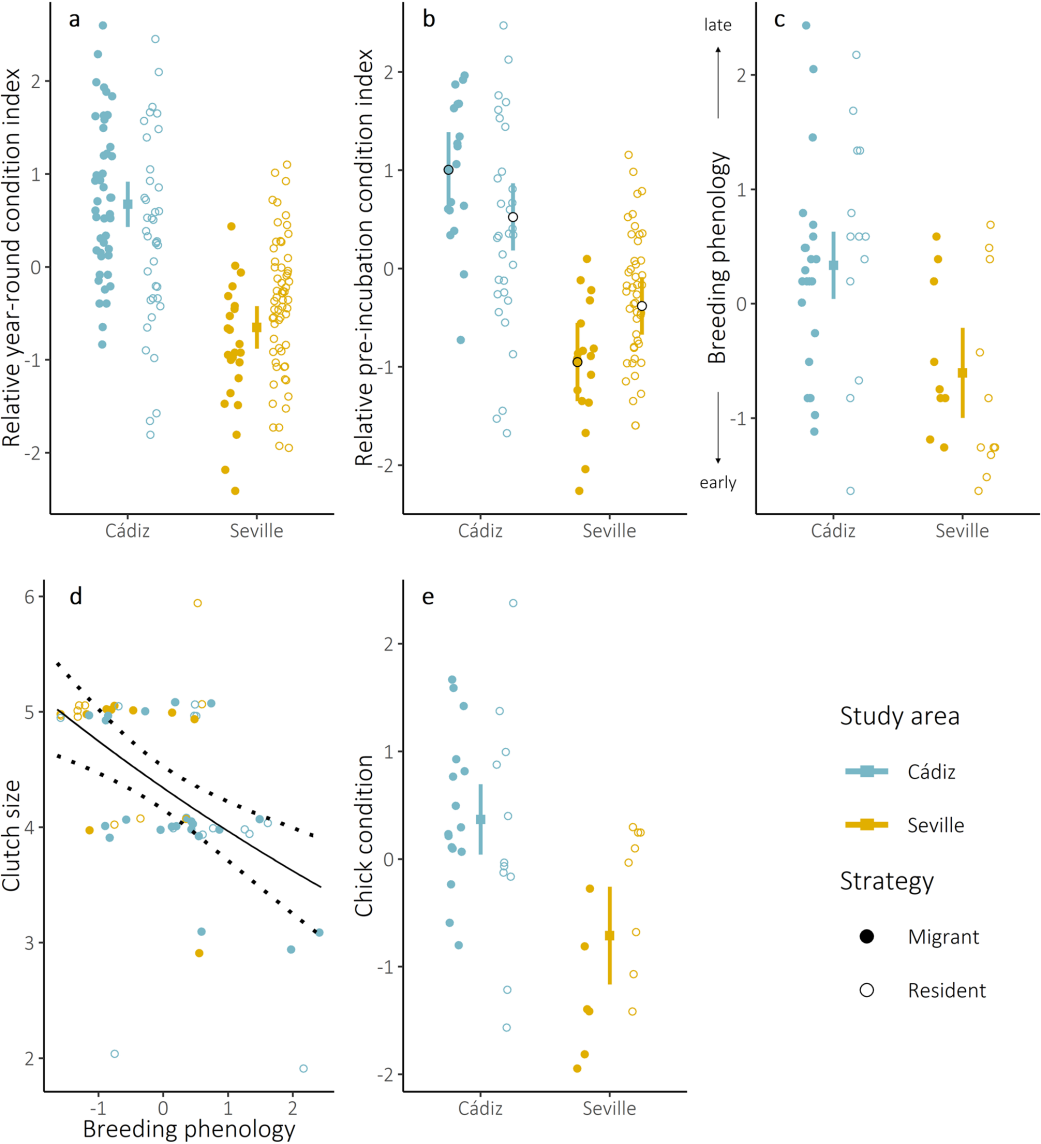
Effects of migratory strategy, breeding site area and breeding phenology on fitness parameters, according to the final model for each analysis. **(a,b)** Relative year-round and pre-incubation condition of adult lesser kestrels captured in the two study areas. Migratory strategy of individual was not an important predictor of year-round condition, but was an important predictor of pre-incubation condition. **(c)** Breeding phenology (scaled first egg date) of lesser kestrel clutches in the two study areas. **(d)** Lesser kestrel clutch size did not vary with study area, but decreased over the season. **(e)** Chick condition from lesser kestrel clutches in the two study areas. Migratory strategy of clutch parent was not an important predictor of breeding phenology, clutch size or chick condition. Points with error bars (Figures a-c and e) or solid line with surrounding dashed lines (Figure d) represent model-predicted means and associated 95% confidence intervals. All continuous variables were scaled and centred via z-score transformation.

Analysis of year-round condition values showed no enduring effect of migratory strategy, but did show an effect of study area (the two colony locations), indicating that birds at Cádiz were consistently in better condition than those at Seville (Fig. 1a).

For condition values measured during the pre-incubation period (see Fig. 3), the most parsimonious model retained the interaction between migratory strategy and study area (Fig. 1b), indicating higher condition in residents compared to migrants in Seville. However, a post-hoc multiple comparisons test was not significant (p = 0.09), suggesting uncertainty around this effect (Supplementary Table S2). Regardless of strategy, birds at Cádiz were in better condition than those in Seville during the pre-incubation period (p < 0.01, Fig. 1b, Supplementary Table S2).

### Breeding success

The most parsimonious model for breeding phenology (first egg date) retained study area as the sole predictor, with lesser kestrels at Cádiz nesting later than at Seville (Fig. 1c). First egg date in turn was the sole important predictor of clutch size, with clutch sizes decreasing over the course of the season (Fig. 1d). Study area was the only predictor in the best model for mean clutch chick condition, with Seville birds having chicks in lower condition than those at Cádiz (Fig. 1e). For all remaining productivity analyses (number of fledglings, nest outcome, fledging probability), the null models were the most parsimonious.

The final models of each analysis are summarised in Supplementary Table S3.

## Discussion

We found no evidence for carryover effects of long-distance migration on phenology, reproductive success or year-round body condition. We identified differences in adult body condition, breeding phenology, clutch size and chick condition between study areas, and weak evidence for short-term carryover effects of migratory strategy on body condition in one study area, with residents in better condition than migrants during the pre-incubation period.

Breeding phenology was similar between migrants and residents, contrary to other studies reporting an association between earlier-breeding and shorter migratory distances^13,28,29^. Migrant lesser kestrels typically return by mid-February^30^, significantly earlier than the start of the breeding season (mid-April), which varies depending on the emergence dates of large invertebrate prey^31^. Residents therefore do not necessarily experience breeding-suitable conditions any earlier than migrants in this species, meaning both strategies may yield similar opportunities to assess the optimal time to commence breeding.

Despite the stark differences in migratory strategy, we also found no significant differences between migrants and residents in clutch size, number of fledglings, nest outcome, fledging probability or chick condition. This may in part be explained by sampling limitations, as we were able to determine the migratory strategy of both parents in only a few cases, and were therefore only able to examine the association between a clutch and the strategy of one parent (see “Methods”). As there is no evidence for phenological differences between migrants and residents, nor observed evidence for assortative mating (of ten known-strategy pairs, three were mixed-strategy), it is possible that carryover effects in these clutches were moderated by a counterbalancing effect of migrant-resident pairs. Carryover effects of migratory strategy on breeding parameters have elsewhere been shown to be strongest where there is a multiplicative effect of matched-strategy pairs^32^. Alternatively, carryover effects of migratory strategy may only manifest later in the season; we did not, for instance, assess post-fledging condition or survival. It may also be the case that, where migratory strategy influences reproductive fitness, it does so to such an extent that individuals simply forego breeding (or do not survive to breed), and are therefore not detected or included in the breeding analysis. Finally, that we do not detect an effect of migratory strategy on productivity may simply indicate that both migrants and residents face different but approximately balanced fitness costs^14^ associated with winter experience (trans-Saharan migration versus enduring harsh winters), ultimately resulting in equal allocation of resources to reproduction.

**Figure 2:**
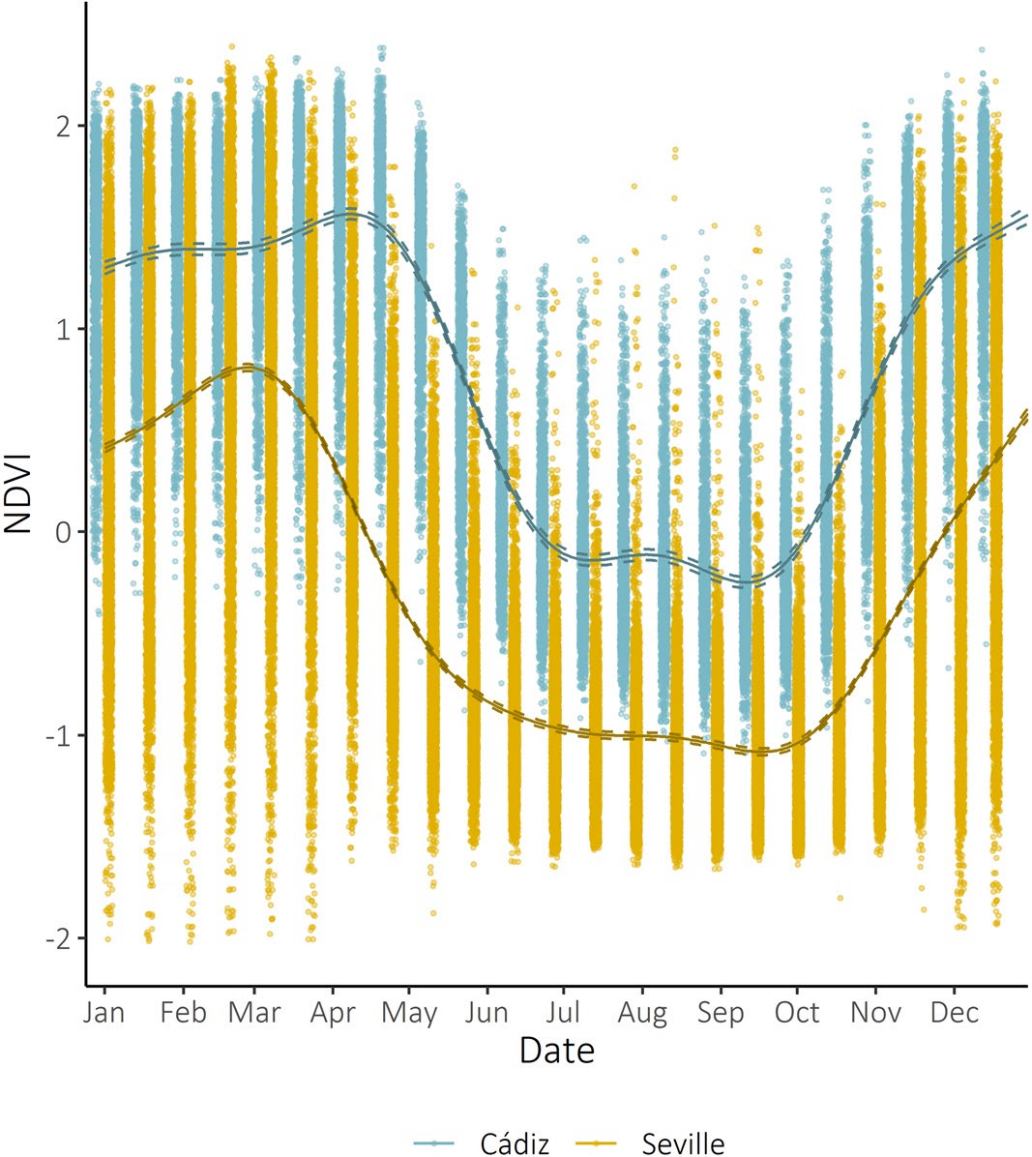
Change in relative primary productivity (scaled NDVI) across the calendar year in the two study areas. We used data from Terra MODIS^66^ and CORINE Land Cover 2012^67^ to extract NDVI values (2014–2018) for each 250-m^2^ pixel classified as suitable foraging habitat (Supplementary Table S4) within a 3-km radius of each colony^68^. We used GAMMs to model NDVI against date smoothed with penalised regression splines, interacting with colony area, with pixel ID as a random effect. The interactions between date and the two levels of study area were significant in the model (p < 0.0001).

Variation in breeding phenology was, to some extent, explained by study area, with birds in Cádiz breeding later than those in Seville (raw mean ± SD first egg date: Cádiz: 28 April ± 10, Seville: 17 April ± 10). Local habitat conditions may play an important role in determining lesser kestrel breeding phenology^31^. The Normalized Difference Vegetation Index (NDVI), considered indicative of food availability for insectivores^33^, was consistently higher in Cádiz than in Seville, and peaked later there (Fig. 2). If NDVI correlates with invertebrate abundance, the later NDVI peak at Cádiz could explain the delayed breeding at that site. This aligns with the patterns observed in nineteen Palaearctic migrant species^2^, where conditions on breeding grounds have a greater effect on breeding phenology than wintering conditions.

Although our results suggest breeding phenology to be the main driver of variation in clutch size—with broods getting smaller over the breeding season, as is commonly found in avian populations^5^—the relationship between breeding phenology and colony area means it is difficult to tease apart the extent to which clutch size is driven by breeding phenology or effects of local breeding conditions. Number of fledglings, nest outcome and probability of fledging were all similar between the two study areas, but chick condition was slightly higher in Cádiz than in Seville (raw mean ± SD chick condition (mass/P8): Cádiz: 2.93 ± 1.10, Seville: 1.86 ± 0.59). Adult individuals in Cádiz also showed better year-round body condition, regardless of migratory strategy, again highlighting the importance of local breeding-site conditions for individual fitness.

We found some statistical support for an effect of migratory strategy on pre-incubation body condition, with resident individuals showing better relative body condition than migrants in the Seville area (Fig. 1b). This may suggest a short-lived carryover effect, with long-distance migration being more costly than residency during the studied period. We might expect this result if migration is a conditional strategy^19,20^ where only high-quality individuals are able to endure winter north of the Sahara (e.g. ^34^). This pattern also aligns with the theory that ongoing environmental change may be disproportionately detrimental to long-distance migrants, increasing relative costs. While not affecting subsequent breeding success, this short-lived carryover effect could plausibly influence survival, with some low-condition migrants not surviving to the subsequent breeding season^11,35^.

No differences were found in year-round body condition between migrants and residents, potentially suggesting that any short-lived carryover effect is counterbalanced by the energetic pressures of reproduction, or buffered by increased food availability during the breeding season. Elsewhere, spring migratory conditions have been found to compensate for the carryover effects of winter conditions on post-winter body condition, with no subsequent effects on breeding success^36^.

It is notable that the potential effect of migratory strategy on pre-incubation adult body condition appeared relevant only in Seville, and not Cádiz, indicating that conditions at the breeding site may override migratory carryover effects. Conditions on breeding grounds have elsewhere been linked to differential manifestations of carryover effects^9,37^; if migration has a greater negative effect on body condition than residency, it is feasible that migrants breeding in more productive habitats recover their condition more quickly upon arrival than individuals in poorer habitats.

Regional differences in the effect of migratory strategy on pre-incubation condition could also arise if there were strong migratory connectivity at this scale, such that migrants from Seville had a distinct and more costly migratory experience than migrants from Cádiz. However, given the proximity of the two areas, strong connectivity seems unlikely. In 2007, a single roost of over 28,000 lesser kestrels was observed in Senegal, representing 30–50% of the western European population^38^, and geolocator studies from elsewhere in Iberia indicate a high degree of spatial aggregation in winter^30,39^. Finally, we might expect regional variation in the effects of migratory strategy if partial migration is both condition- and density-dependent^19^, with different mechanisms underpinning migration and residency in the two areas^40,41^.

Sample size limitations may have influenced our overall power to detect carryover effects of migratory strategy, although our main finding—that breeding site effects are of greater relevance to fitness than migratory strategy—concerns the relative magnitude of effects, rather than their absolute size. Carryover effects of strategy on breeding may be buffered in broods with mixed-strategy parents, something we were unable to analyse. That we were only able to assess the effect of migratory strategy of one clutch-parent may have undermined our capacity to detect carryover effects on breeding parameters. We did not examine survival, recruitment or population trends—strong effects of migratory behaviour on fitness could therefore be concealed if they operate largely on survival parameters rather than reproductive success. Similarly, knock-on effects of reproductive effort and body condition may only be reflected in subsequent mortality. Measuring survival in conjunction with post-fledging survival and/or recruitment would shed further light on population-level effects of migratory variability.

## Conclusions

Despite marked differences in the wintering experiences of migrant and resident lesser kestrels, carryover effects of migratory strategy were limited and idiosyncratic, with conditions on the breeding grounds being of greater relevance for adult and chick body condition. We hypothesised that anthropogenic change could be having a disproportionate effect on migrants, and thereby disrupting the balance in fitness benefits of each strategy. We found little evidence to support this, suggesting that costs of migration associated with exposure to anthropogenic impacts may be counterbalanced by costs experienced by resident individuals, such as variability in winter conditions. This apparent parity of fitness between the two strategies is in accordance with theory on the evolutionary stability of partially migratory populations^14^. Detailed information on adult survival and chick recruitment may facilitate more comprehensive understanding of the interactions between migratory strategies, breeding conditions and demographic effects.

## Methods

### Ethics statement

All bird handling and fieldwork protocols were conducted according to the relevant national and institutional regulations on animal welfare, and were approved by the Junta de Andalucía: Dirección General de Gestión del Medio Natural y Espacios Protegidos (Ntra. Ref: 2016107300003028/IRM/MDGC/mes) and the University of East Anglia Animal Welfare and Ethical Review Board.

### Study system

The lesser kestrel is a small, colonial raptor^42^, breeding largely in abandoned agricultural structures or in towns^43^, with a largely insectivorous diet^44^. We studied four colonies in two regions of Andalucía, southern Spain: three in the province of Seville, breeding in abandoned agricultural buildings on the border between Doñana National Park and surrounding arable farmland in the Guadalquivir basin (37°05′N 6°19′W), and a fourth in the province of Cádiz, breeding in a church tower in Los Barrios town (36°11′N 5°30′W).

### Monitoring breeding phenology and success

We monitored breeding parameters in 2014–2018 (Seville colonies) and 2014–2017 (Cádiz colony), visiting colonies weekly to monitor all accessible cavities and collect data on nesting phenology, clutch size and fledging success (number of chicks aged > 20 days per nest); chicks were also ringed and measured prior to fledging. First egg dates were either observed directly or back-calculated as 32 days prior to the first hatch date^45^. Our data indicate that wing growth of lesser kestrels is linear between the ages of 14 and 30 days, with non-linear growth likely to occur after day 30 (Supplementary Figure S2, see also^46,47^). If neither first egg date nor first hatch date were directly observed (n = 17 clutches), we therefore estimated these using a linear mixed-effects model of wing chord measurements against known-ages of chicks measured in 2018 (n = 96, marginal R^2^ = 0.89) (see Supplementary Figure S2). We deemed a nest successful if at least one bird reached an age greater than 20 days.

### Determining migratory strategies

We used a combination of wintering observations, geolocator data, and feather isotope analysis to determine individual strategies (migrant or resident). At each colony, we captured adults via opportunistic captures during nest visits, mist-netting close to roost sites, spring-traps baited with insect or small mammal prey, and nocturnal visits to colonies. All individuals were colour-ringed, aged and sexed according to plumage features. We measured mass, wing chord and eighth descendant primary (P8) length at each capture, and collected a c. 1 cm^2^ section of vane from the trailing edge of the winter-grown ninth or tenth primary (P9/P10) feather of each adult bird once per calendar year for isotope analysis. We classified migratory strategies of each individual in each year (henceforth ‘bird-years’), and related these strategies to metrics of body condition and breeding success in the following season.

#### Direct observations

Colonies were visited weekly during the winter (Nov–Jan) from 2013 to 2018 to identify residents through resightings. Any individual detected in Seville or Cádiz between 01 November and 15 January was considered a resident bird-year for the breeding season immediately following (date thresholds represent a two-week buffer around our earliest observations of known migratory individuals). Additionally, one bird was a confirmed migrant from an opportunistic resighting in Senegal in 2017.

#### Geolocators

We deployed 36 geolocators (British Antarctic Survey model Mk14, 1.5 g, attached on Teflon harnesses as back mounts) during the 2014 and 2015 breeding seasons, of which 16 were subsequently recovered and 13 provided adequate data. Geolocators were pre- and post-calibrated for 7 days, and analysed using the ‘FLightR’ package following^48,49^. Individual twilights were excluded if light–dark transitions were erratic 2 h either side of the twilight. Individuals were assumed to be resident if they showed no fixes south of 36°N between 01 November and 15 January, and migratory if they showed at least two fixes south of 23°N within that period.

#### Feather isotopes

We used δ^13^C values (ratios of the stable isotopes ^13^C to ^12^C) of winter-grown flight feathers P9/10^50^ to identify migrant individuals (see Supplementary Information: Isotope analysis methods). As some individuals moult P9/P10 prior to migration^51,52^, birds with an Iberian isotopic signature (lower δ^13^C values) for winter-grown feathers cannot be assumed to be resident, as they could have moulted the feather on the breeding grounds prior to migrating. We therefore only used isotope values to identify migrants, not residents.

We analysed δ^13^C values from the feathers of 276 bird-years, to which we added isotope data from an additional 128 bird-years collected from other colonies in the same region (37°21′N 5°13′W). We classified any feather with a δ^13^C value higher than − 20 ‰ to come from a migrant individual, deeming a δ1^3^C value of − 20 ‰ a conservative buffer around the clustering of lower δ^13^C values representing feathers grown on the breeding grounds, and excluding all known residents (Supplementary Fig. S3). This cut-off also aligns with the δ^13^C feather isoscape for Africa created by Hobson and colleagues^53^. We expect at least 70% individuals in the study populations to be migratory^27,54^, and this conservative cut-off likely excludes a number of migrants from our analysis, but as we were not assessing prevalence of strategies, we analysed only those individuals we could confidently classify as long-distance migrants. We also undertook a sensitivity analysis of the -20 ‰ cut-off value, running all analyses on two additional datasets, with isotope-defined migratory status created according to either a more conservative or less conservative δ^13^C cut-off (± 0.5 ‰). Altering this value did not change our results for any analyses, indicating the results reported are robust to variations in this threshold—see Supplementary Materials: Isotope sensitivity analyses.

### Condition metrics

**Figure 3:**
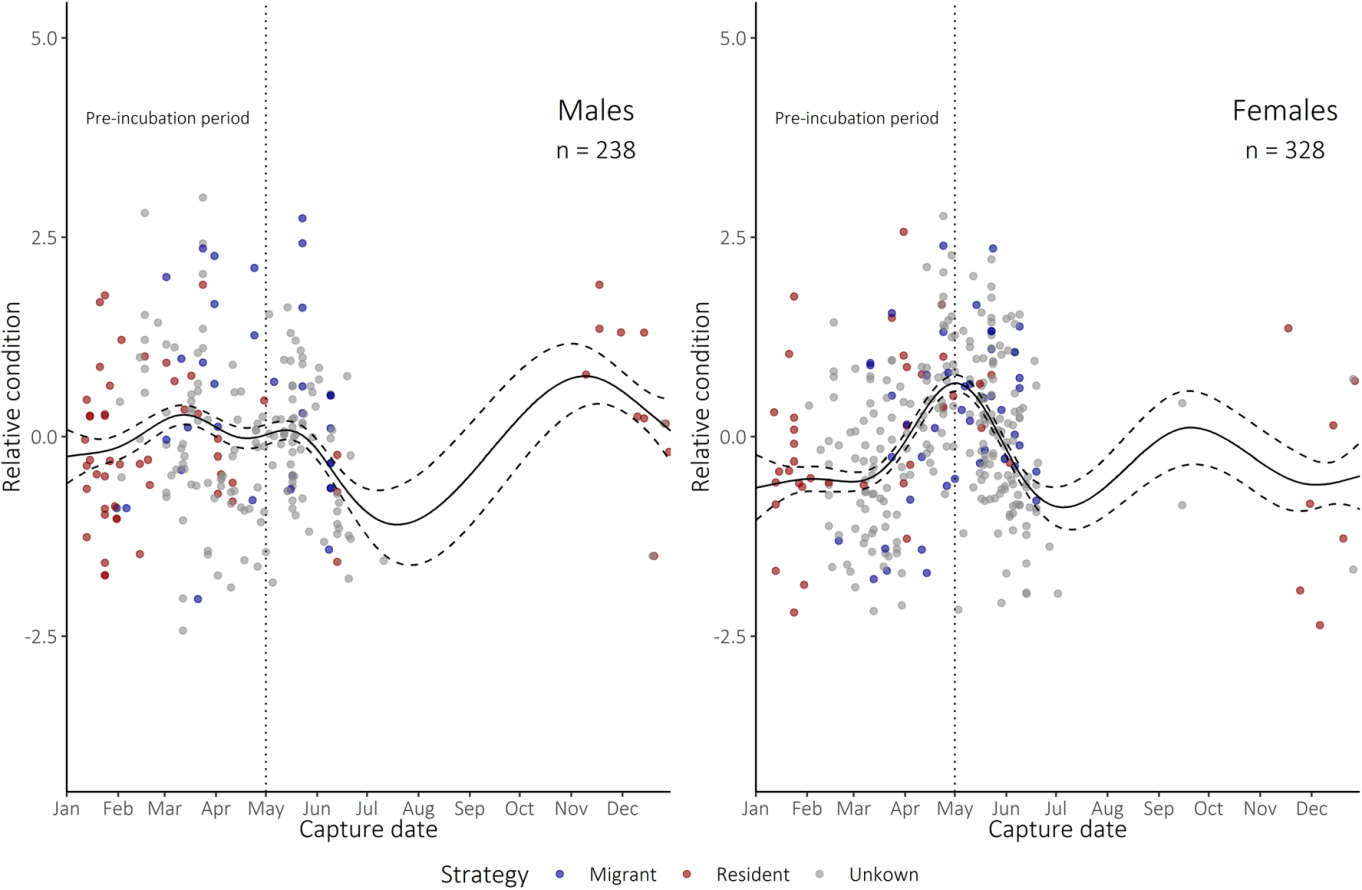
Relative body condition (scaled mass/P8 length) of adult lesser kestrels caught throughout the year. Black lines indicate model-predicted mean condition, dashed line indicates associated standard error. Colours indicate migratory strategy during the winter preceding the breeding season in the calendar year of capture. Captures occurring prior to the date indicated by the dotted line (May 01) were used in the pre-incubation condition analysis.

To estimate adult condition at capture, we divided mass by P8 length to distinguish dynamic body condition from structural size^55^. We then developed an index of bird condition relative to the population average, controlling for sex and stage of the annual cycle, using generalised additive mixed-effect models (GAMMs) with penalised regression splines, adding bird identity as a random effect to account for recaptures (n = 566 captures across all years of data—Fig. 3). We took the residuals from these models as our index of body condition relative to the population-wide mean condition for individuals of that sex at that date. We created GAMMs using the package ‘gamm4′^56^, with default selection of smoothing parameters.

For chick condition, we again divided mass by P8 length to account for chicks having been measured at different ages, and averaged this condition across siblings to create a single mean chick condition for each brood.

### Statistical analysis

For all final analyses, we excluded data from 2018, as there was an imbalance in sampling effort between the two study regions (Supplementary Fig. S1).

#### Adult body condition

We modelled the relationship between relative adult body condition and migratory strategy using GLMMs with a Gaussian distribution, including bird identity as a random effect, using the package ‘lme4′^57^. We also included capture date, study area and sex as fixed effects, and allowed for an interaction between strategy and study area (Cádiz vs. Seville) to examine whether carryover effects differed between areas. Because short-term carryover effects of migratory strategy might be strongest immediately after pre-breeding migration, we repeated the analysis using only condition measures taken prior to the peak in mean female condition (02 May, Fig. 3), corresponding with the onset of incubation—henceforth referred to as the ‘pre-incubation period’—considering this to represent the point after which body condition becomes more strongly affected by breeding effort than by the preceding winter. We conducted a post-hoc multiple comparison test on the resulting final model, using the package ‘multcomp’^58^.

#### Breeding parameters

We analysed the influence of migratory strategy on breeding phenology (first egg date), clutch size, number of fledglings, nest outcome (success or failure), fledging probability (proportion of eggs successfully fledged) and chick condition. Study area was included in all models. For all productivity analyses barring chick condition, we also considered first egg date as a fixed effect, hypothesising that phenology may affect productivity. For the chick condition analysis we had a relatively small sample size (n = 41, Supplementary Fig. S1), within which first egg date was confounded with study area, and was therefore excluded.

We analysed breeding parameters for all adults of known migratory strategy for which there was clutch data. For clutches where the migratory strategy of both parents was known (n = 10), one parent was excluded at random to avoid duplicating these clutches in the dataset (see Supplementary Fig. S1 for sample sizes). We used generalised linear models with a Gaussian distribution for phenology and chick condition, and Poisson distribution for number of fledglings, with a log link function. For clutch size we used a Conway-Maxwell Poisson model^59^ to account for underdispersion, using automatic estimation of the under-dispersion parameter (*ν*)^60,61^. We used a binomial distribution with logit link function for nest outcome, and modelled fledging probability (weighted by clutch size) using a quasibinomial distribution—to handle overdispersion (pertinent for binomial models using proportion data)— and logit link function.

In all instances, we followed a model-theoretic approach^62^, creating a subset of reduced models from each global model, then ranking these by Akaike’s Information Criterion adjusted for small sample size (AICc) to determine the best-fitting models, using the package ‘MuMIn’^63^. We used quasi-AICc (QAICc) values for the fledging probability analysis, as AIC values cannot be calculated for models with quasi-distributions. Where we had multiple competitive models (within Δ2 (Q)AICc units of the highest-ranked model), we selected the model with the fewest parameters within the Δ2 subset as our final model^62^, as model-averaging is not feasible for model sets including interaction terms^64^.

We scaled and centred all continuous variables via Z-score transformations, and carried out all analyses in R version 3.5.0^65^.

## Supplementary Information

Supplementary Information.

Supplementary Dataset.

## Data Availability

The data underpinning these analyses and the relevant R code are included in the Supplementary Information.
