# Supplementary material for: Carryover effects of long-distance avian migration are weaker than effects of breeding environment in a partially migratory bird
Source: Sci Rep. 2021 Jan 13;11:935. doi: 10.1038/s41598-020-80341-x (PMC7807013; doi:10.1038/s41598-020-80341-x)
Supplement: Supplementary file 1 — Supplementary Information. [file 41598_2020_80341_MOESM1_ESM.pdf]

Carryover effects of long-distance avian migration are weaker than effects of breeding environment in a partially migratory bird

Claire Buchan*, James J. Gilroy, Inês Catry, Javier Bustamante, Alina D. Marca, Philip W. Atkinson, Juan Miguel González, Aldina M. A. Franco

* Corresponding author: c.buchan@uea.ac.uk

This document consists of the following supplementary materials relating to the above manuscript

- Isotope analysis methods
- Supplementary Tables
- Supplementary Figures
- Isotope sensitivity analyses
- R Code
- References

Isotope analysis methods

Distinct photosynthetic processes used by different plant species result in varying ratios of the stable isotopes ^{13}C to ^{12}C (expressed as $\delta^{13}C$). The spatial distributions of these species dictate geographically varying values of $\delta^{13}C$: the photosynthetic pathway used by plant species adapted to hot, dry climates (the C_4 pathway) results in higher $\delta^{13}C$ values in regions where these plants are more prevalent than elsewhere[1,2]. Feathers incorporate (via trophic interactions) the approximate isotopic signature of the environment in which they are grown; feather $\delta^{13}C$ values can therefore be used to infer the broad location of this environment[1] – see [2] for isoscape, subsequently adapted specifically for feathers (based on +2‰ accumulation of $\delta^{13}C$)[3].

Lab work was carried out at the University of East Anglia (UEA); each sample was analysed for carbon and nitrogen in the year of its collection (though only carbon values were used for strategy determination). Feather samples were rinsed in a 2:1 chloroform: methanol solvent to clean surface oils and air-dried for 48 hours. From each cleaned sample, a cutting of approximately 0.2 cm^2 was encapsulated in tin foil, subsequently combusted in a Costech elemental analyser interfaced with a Thermo Scientific Delta XP continuous flow mass spectrometer. We simultaneously analysed an internal reference material (casein) and laboratory internal standard material (collagen). Stable isotope ratios are measured in parts per thousand (per mille: ‰) and expressed in δ notation, relative to an international standard, which for carbon is the Vienna-Pee Dee Belemnite carbonate (VPDB) and for nitrogen is atmospheric nitrogen (AIR N_2). The precision of carbon composition varied from 0.1–0.3 ‰, while the precision of nitrogen composition varied from 0.1–0.4 ‰.

Carryover effects of long-distance avian migration are weaker than effects of breeding environment in a partially migratory bird

Claire Buchan, James J. Gilroy, Inês Catry, Javier Bustamante, Alina D. Marca, Philip W. Atkinson, Juan Miguel González, Aldina M. A. Franco

Supplementary Tables

Supplementary Table S1 – Total bird-years (individual birds within a specific year) of all ages with known migratory strategies, as identified through three main strategy determinants: observations, geolocator data and feather isotope signatures.

Resident strategy determinant	Number identified	Total residents (bird-years)
Observed resident	89	
Geolocator resident	4 (of which 1 additional to those observed in winter)	90
**Migrant strategy determinant**	**Number identified**	**Total migrants (bird-years)**
Observed migrant	1	
Geolocator migrant	9	61
Isotope signature ($\delta^{13}C$ > -20 ‰)	53 (of which 51 additional to geolocator migrants)	

Supplementary Table S2 – Outputs of post-hoc test of multiple comparisons carried out on the final best-ranked GLMM assessing the effect of migratory strategy and study area on adult pre-hatching condition. See Fig. 1b.

Pairwise comparison			Estimate	SE	z value	Pr(>|z|)
Cádiz resident	–	Cádiz migrant	-0.48	0.26	-1.84	0.25
Seville migrant	–	Cádiz migrant	-1.96	0.28	-6.94	< 0.001
Seville resident	–	Cádiz migrant	-1.39	0.25	-5.65	< 0.001
Seville migrant	–	Cádiz migrant	-1.48	0.27	-5.51	< 0.001
Seville resident	–	Cádiz resident	-0.91	0.23	-3.95	< 0.001
Seville resident	–	Seville migrant	0.57	0.25	2.32	0.09

Claire Buchan, James J. Gilroy, Inês Catry, Javier Bustamante, Alina D. Marca, Philip W. Atkinson, Juan Miguel González, Aldina M. A. Franco

Supplementary Table S3 – Effect size summary for each analysis, with coefficient estimates (± standard error) for parameters retained in the relevant final model. The reference categories for the two two-level categorical variables (Strategy and Study Area) are Migrant and Cádiz, respectively. Parameters marked with a dash were present in the global model for the relevant analysis but not retained in the most competitive model; greyed out parameters were not included in the relevant global model.

n	Figure	Response	Model structure:					
			Strategy (Resident)	Study area (Seville)	Strategy(Resident)*Study area(Seville)	First egg date	Capture date	Sex
170	1a	Year-round body condition	-	-1.33 (±0.17)	-		-	-
112	1b	Pre-incubation body condition	-0.48 (±0.26)	-1.96 (±0.28)	1.05 (±0.35)		-	-
56	1c	Breeding phenology	-	-0.94 (±0.25)	-			
56	1d	Clutch size	-	-	-	-0.81 (±0.20)		
56	-	Fledglings	-	-	-	-		
56	-	Nest outcome	-	-	-	-		
56	-	Fledglings per egg	-	-	-	-		
41	1e	Chick condition	-	-1.08 (±0.29)	-			

Supplementary Table S4 – CORINE Land Cover (CLC) habitat categories deemed suitable for lesser kestrel foraging. Pixels classified as these habitat categories within a 3-km radius of a colony were used for the assessment of study area primary productivity (NDVI). See [4] for detailed land cover definitions.

Land cover category	CLC Code
Roads and rail networks and associated land	122
Non-irrigated arable land	211
Permanently irrigated arable land	212
Pastures	231
Annual crops associated with permanent crops	241
Complex cultivation patterns	242
Land principally occupied by agriculture, with significant areas of natural vegetation	243
Agro-forestry areas	244
Natural grassland	321
Inland marshes	411

Carryover effects of long-distance avian migration are weaker than effects of breeding environment in a partially migratory bird

Claire Buchan, James J. Gilroy, Inês Catry, Javier Bustamante, Alina D. Marca, Philip W. Atkinson, Juan Miguel González, Aldina M. A. Franco

Supplementary Figures

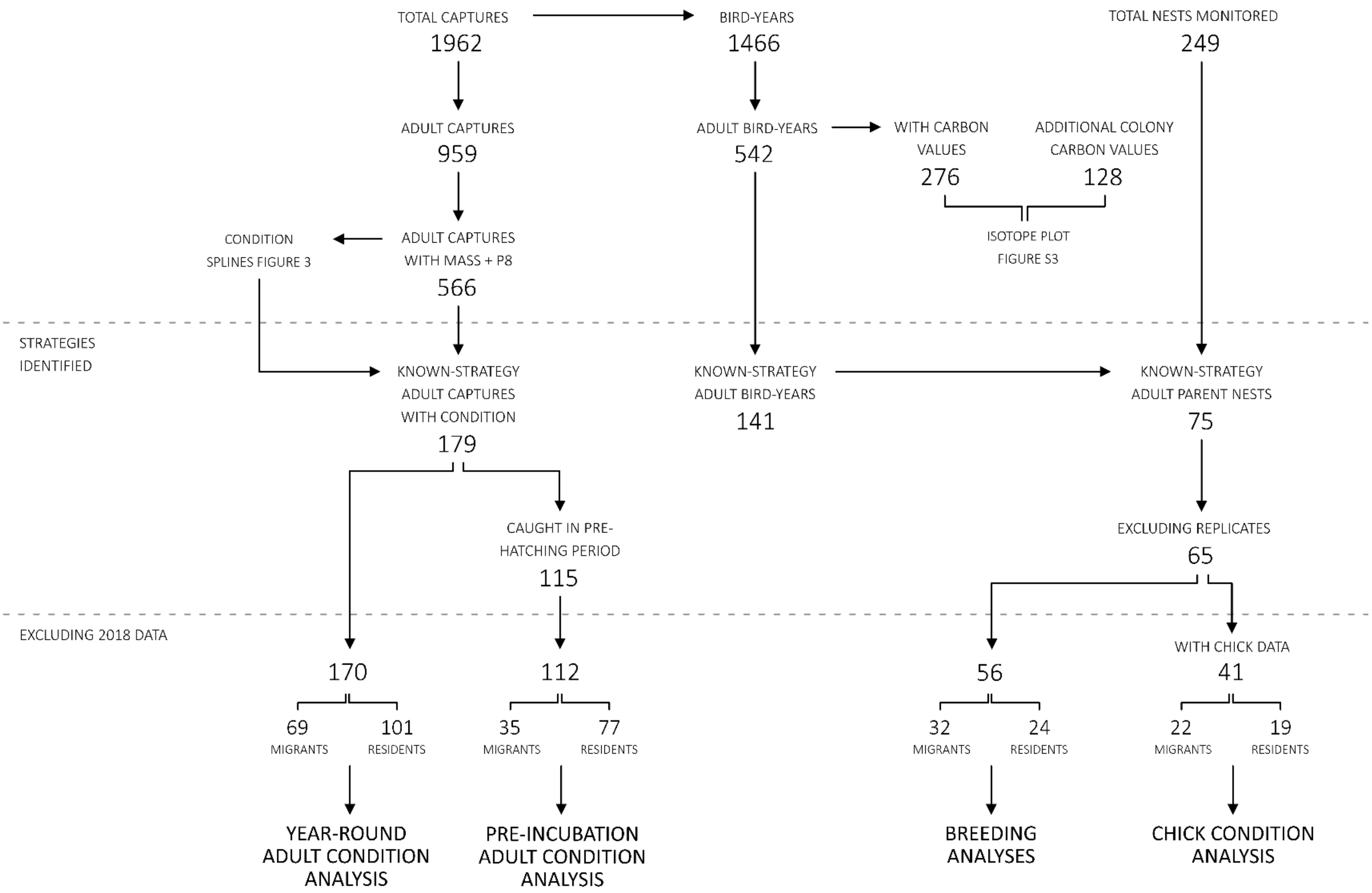

Supplementary Figure S1 – Visualisation of data-filtering and resulting sample sizes for each analysis.

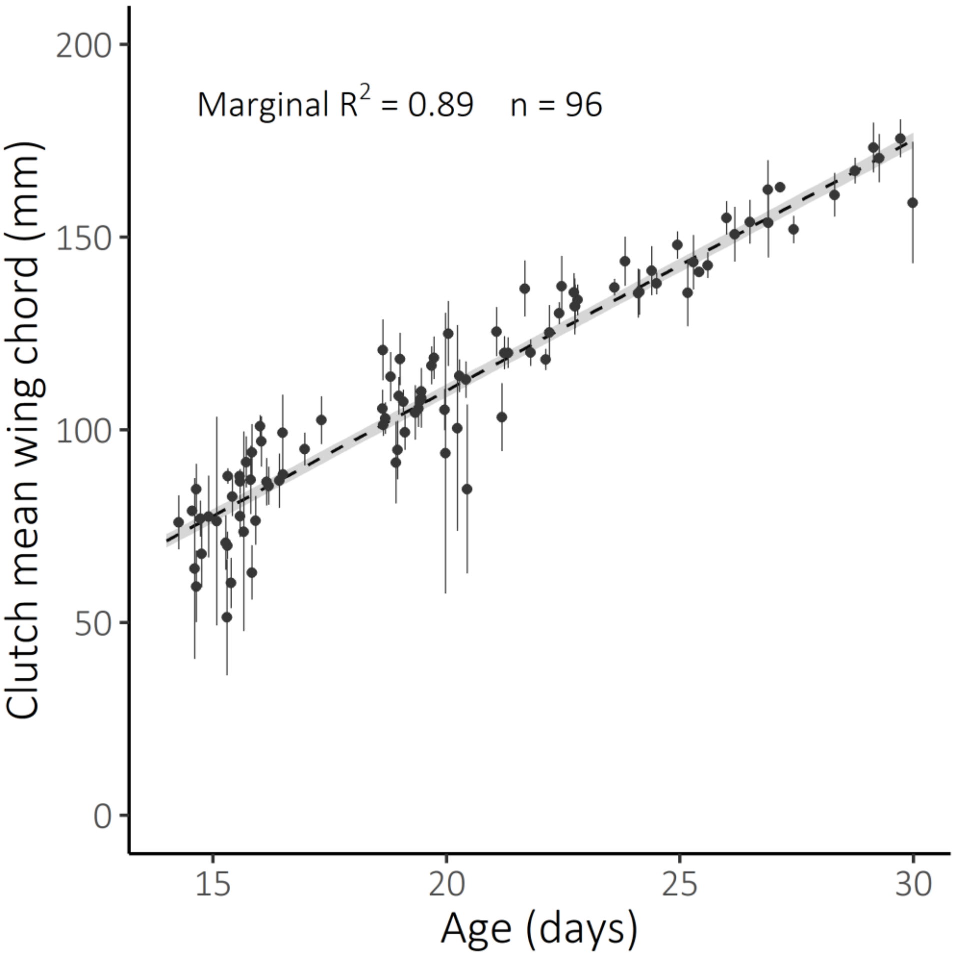

Supplementary Figure S2 – Relationship between clutch age (days since first hatch date) and chick wing chord measurements taken in 2018 from the Seville colonies. Points represent mean clutch wing chord, with associated standard error shown by vertical lines. Black dashed line represents the effect of age on mean clutch wing chord as predicted by a linear mixed-effects model with clutch as a random effect; grey ribbon represents standard error. The formula of this linear mixed-effects model (y = -19.8 + 6.5x) was used to calculate clutch age for those clutches for which neither first egg date nor first hatch date were observed (n=17). Clutch age is therefore given by: (wing chord + 19.8)/6.5.

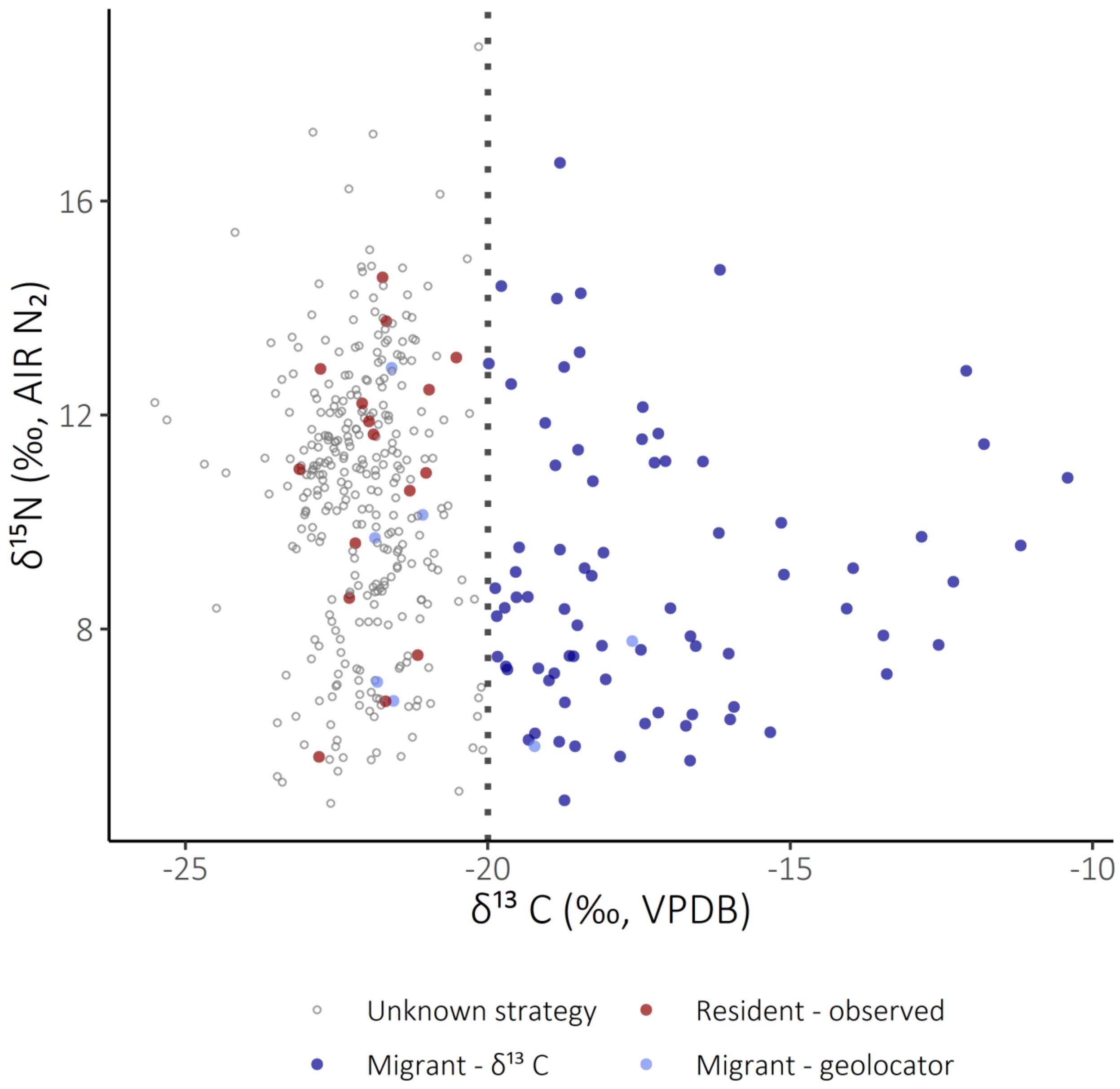

Supplementary Figure S3 – Feather isotope values (δ^{15}N plotted against δ^{13}C) collected from individuals of known and unknown migratory strategies (n=404). Point colour indicates strategy and strategy-determinant method. Dotted line indicates the δ^{13}C migratory strategy-determinant cut-off – individuals with δ^{13}C values higher than -20 ‰ were determined to be migrants. Individuals with values lower than this cut-off could either be residents or migrants that moulted prior to migration – for this reason, individuals with values lower than -20 ‰ were classed as unknown strategy unless directly observed as a resident or geolocated as a migrant. Neither the single observed migrant nor the four geolocator residents had associated feather isotope data; these are therefore not represented on this plot.

Isotope sensitivity analyses

The results reported in the manuscript were conducted on a dataset in which isotope-defined migrants were identified according to a $\delta^{13}C$ cut-off value of -20 ‰ (Supplementary Fig. S3). To assess the sensitivity of our results to the cut-off value, we repeated all analyses on two additional datasets, one with a less conservative cut-off of -20.5 ‰ (and therefore a greater number of isotope-defined migrants), and one with a more conservative cut-off of -19.5 ‰ (fewer isotope-defined migrants – Supplementary Fig. 4). The alteration in cut-off value by ±0.5 ‰ was decided based on the highest $\delta^{13}C$ signature of a known resident bird (-25.2 ‰).

In both additional analyses, all final models retained the same parameters as the final models in the original analyses, with comparable coefficient estimates and standard errors (Supplementary Tables S5+S6) – indicating that our results are robust to variations in the cut-off value for defining migrants from $\delta^{13}C$.

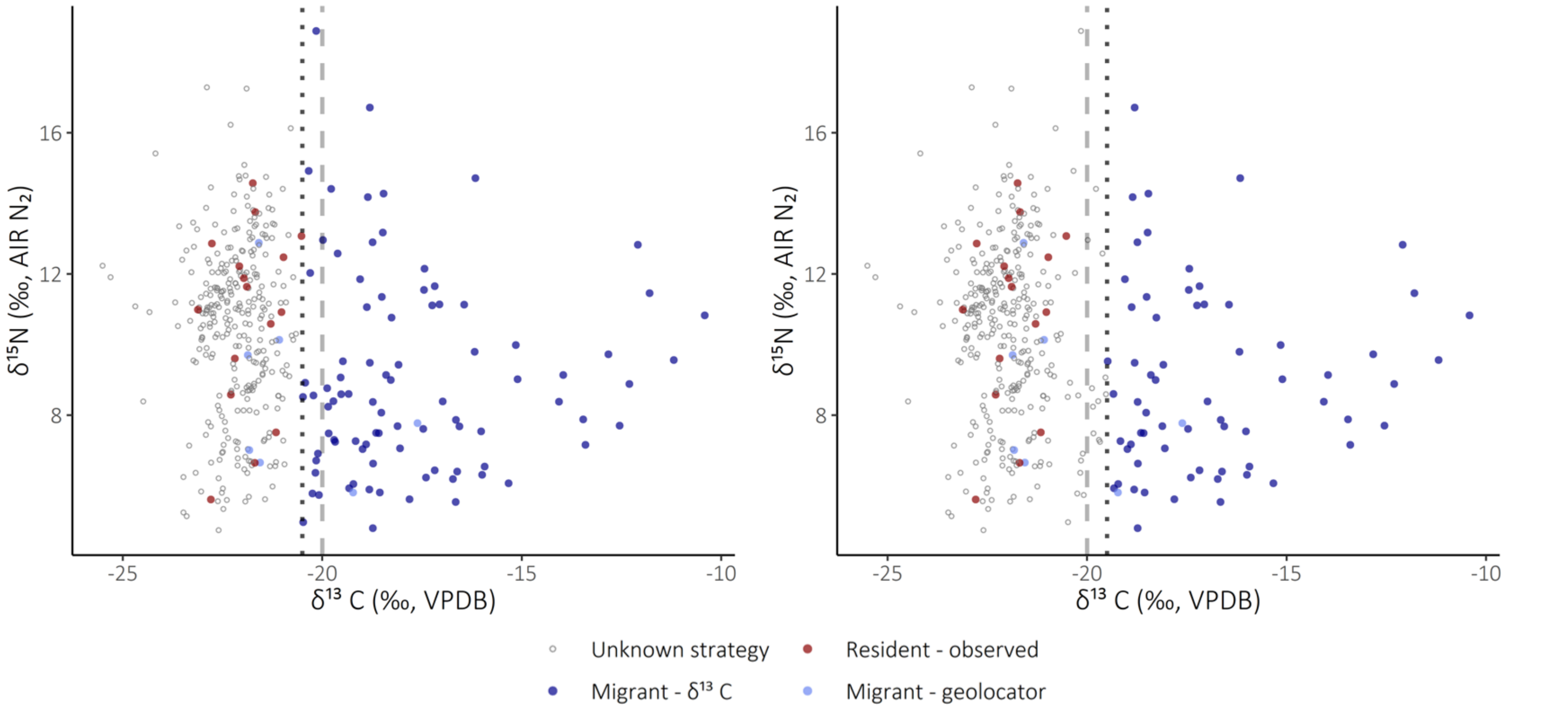

Supplementary Figure S4 – Visualisation of the shift in the $\delta^{13}C$ migratory-determinant cut-off value from the original (Supplementary Fig. 3), showing the migrant datapoints gained (L) and lost (R) in datasets underlying the two sensitivity analyses. Feather isotope values ($\delta^{15}N$ plotted against $\delta^{13}C$) collected from individuals of known and unknown migratory strategies (n=404). Point colour indicates strategy and strategy-determinant method. Dotted line indicates the new $\delta^{13}C$ migratory strategy-determinant cut-off – individuals with $\delta^{13}C$ values higher than -20.5 ‰ (L) or -19.5 ‰ (R) were determined to be migrants. Faint dashed line indicates the original -20 ‰ migratory strategy-determinant cut-off used in the main analysis.

Supplementary Table S5 – Effect size summary for sensitivity analyses conducted on the dataset with isotope-defined migrants identified using a (less conservative) $\delta^{13}C$ cut-off value of -20.5 ‰. Coefficient estimates (± standard error) for parameters retained in the relevant final mode can be compared to those of the main analysis given in Supplementary Table S3. Delta n indicates the change in sample size from the original dataset. The reference categories for the two two-level categorical variables (Strategy and Study Area) are Migrant and Cádiz, respectively. Parameters marked with a dash were present in the global model for the relevant analysis but not retained in the most competitive model; greyed out parameters were not included in the relevant global model.

n	Δ n	Response	Model structure:					
			Strategy (Resident)	Study area (Seville)	Strategy(Resident)*Study area(Seville)	First egg date	Capture date	Sex
178	+8	Year-round body condition	-	-1.19 (±0.17)	-		-	-
113	+1	Pre-incubation body condition	-0.48 (±0.26)	-1.94 (±0.28)	1.03 (±0.36)		-	-
63	+7	Breeding phenology	-	-0.80 (±0.24)	-			
63	+7	Clutch size	-	-	-	-0.78 (±0.18)		
63	+7	Fledglings	-	-	-	-		
63	+7	Nest outcome	-	-	-	-		
63	+7	Fledglings per egg	-	-	-	-		
48	+7	Chick condition	-	-0. 88 (±0.28)	-			

Claire Buchan, James J. Gilroy, Inês Catry, Javier Bustamante, Alina D. Marca, Philip W. Atkinson, Juan Miguel González, Aldina M. A. Franco

Supplementary Table S6 – Effect size summary for sensitivity analyses conducted on the dataset with isotope-defined migrants identified using a (more conservative) $\delta^{13}C$ cut-off value of -19.5 ‰. Coefficient estimates (± standard error) for parameters retained in the relevant final mode can be compared to those of the main analysis given in Supplementary Table S3. Delta n indicates the change in sample size from the original dataset. The reference categories for the two two-level categorical variables (Strategy and Study Area) are Migrant and Cádiz, respectively. Parameters marked with a dash were present in the global model for the relevant analysis but not retained in the most competitive model; greyed out parameters were not included in the relevant global model.

n	Δ n	Response	Model structure:					
			Strategy (Resident)	Study area (Seville)	Strategy(Resident)*Study area(Seville)	First egg date	Capture date	Sex
159	-11	Year-round body condition	-	-1.29 (±0.18)	-		-	-
107	-5	Pre-incubation body condition	-0.30 (±0.29)	-1.86 (±0.31)	0.93 (±0.39)		-	-
51	-5	Breeding phenology	-	-0.92 (±0.26)	-			
51	-5	Clutch size	-	-	-	-0.80 (±0.21)		
51	-5	Fledglings	-	-	-	-		
51	-5	Nest outcome	-	-	-	-		
51	-5	Fledglings per egg	-	-	-	-		
37	-4	Chick condition	-	-1.05 (±0.30)	-			

R code

```
library(dplyr)
library(lme4)
library(nlme)
library(mgcv)
library(gamm4)
library(gdata)
library(arm)
library(MuMIn)
library(lmerTest)
library(multcomp)
library(spaMM)
library(LMERConvenienceFunctions)
con<-read.csv("Buchan_adult_condition_dataset.csv", header=T)
prod<-read.csv("Buchan_productivity_dataset.csv", header=T)
# year-round adult condition -------------
fs<-drop.levels(subset(con, sex=="female"))
ms<-drop.levels(subset(con, sex=="male"))
ms$ma.p8<-scale(log(ms$ma.p8))
fs$ma.p8<-scale(log(fs$ma.p8))
fs_gam<-gamm4(ma.p8 ~ s(capday, fx=FALSE, k=-1),family=gaussian,
             random=~(1|code),data=fs)
ms_gam<-gamm4(ma.p8 ~ s(capday, fx=FALSE, k=-1),family=gaussian,
             random=~(1|code),data=ms)
fs$condition<-resid(fs_gam$gam, type="pearson")
ms$condition<-resid(ms_gam$gam, type="pearson")
c<-rbind(fs, ms)
c<-drop.levels(subset(c, res!="UNK"))
summary(c)
yearcon<-lmer(scale(condition)~capday+res*zone+sex+(1|code),
              na.action=na.fail, REML=FALSE,
              data=c)
yearcon_d<-dredge(yearcon)
yearcon_d
yearcon_best<-get.models(yearcon_d,subset=2)[[1]]
summary(yearcon_best)
# pre-incubation adult condition -------------
c_early<-drop.levels(subset(c, capday<122))
summary(c_early)
earlycon<-lmer(scale(condition)~sex+capday+res*zone+(1|code),
               na.action=na.fail, REML=FALSE,
               data=c_early)
earlycon_d<-dredge(earlycon)
earlycon_d
earlycon_best<-get.models(earlycon_d,subset=1)[[1]]
summary(earlycon_best)
c_early$INT<-interaction(c_early$res, c_early$zone)
intmod<-lmer(scale(condition)~-1+INT+(1|code),
             na.action=na.fail, REML=FALSE,
             data=c_early)
summary(intmod)
summary(glht(intmod, linfct = mcp(INT = "Tukey")))
# phenology --------
prod$res<-as.factor(prod$res)
fed<-lm(scFED~res*zone,
        data=prod, na.action=na.fail)
FEDd<-dredge(fed)
FEDd
bestFED<-get.models(FEDd, subset=1)[[1]]
summary(bestFED)
# clutch size ------
clutch<-glm(nbrood~as.vector(scFED)+res*zone,
            data=prod, family=COMPoisson(nu=10), na.action=na.fail)
clutch_d<-dredge(clutch)
clutch_d
clutch_best<-get.models(clutch_d, subset=1)[[1]]
```

```
summary(clutch_best)
# no. fledglings ------
fl<-glm(nfledge~scFED+res*zone,
        family=poisson(link=log), data=prod,
        na.action=na.fail)
fl_d<-dredge(fl)
fl_d
# nest outcome --------
out<-glm(outcome~scFED+res*zone,
         family=binomial, data=prod,
         na.action=na.fail)
out_d<-dredge(out)
out_d
# fledglings per egg ----------
prod$fl.br<-prod$nfledge/prod$nbrood
prop<-glm(fl.br~scFED+res*zone,
          weights=nbrood,
          family=quasibinomial(), data=prod,
          na.action = na.fail)
quasi <- function(...) {
  res <- quasibinomial(...)
  res$aic <- binomial(...)$aic
  res
}
prop_d<-dredge(update(prop, family = quasi), rank=QAIC,chat=summary(prop)$dispersion)
prop_d
# chick condition ------
ch<-drop.levels(prod[!is.na(prod$ch_condition),])
ch$sc_ch<-scale(log(ch$ch_condition))
chcon<-lm(sc_ch~res*zone,
          data=ch, na.action=na.fail)
chcon_d<-dredge(chcon)
chcon_d
con_best<-get.models(chcon_d, subset=2)[[1]]
summary(con_best)
```

References

1. Hobson, K. A., Barnett-Johnson, R. & Cerling, T. E. Using Isoscapes to Track Animal Migration in *Isoscapes: Understanding Movement, Pattern, and Process on Earth Through Isotope Mapping* (eds. West, J. B., Bowen, G. J., Dawson, T. E. & Tu, K. P.) 273–298. (Springer US, 2010).
2. Still, C. J. & Powell, R. L. Continental-Scale Distributions of Vegetation Stable Carbon Isotope Ratios in *Isoscapes: Understanding Movement, Pattern, and Process on Earth Through Isotope Mapping* (eds. West, J. B., Bowen, G. J., Dawson, T. E. & Tu, K. P.) 179–193 (Springer US, 2010).
3. Hobson, K. A. *et al.* A multi-isotope ($\delta^{13}C$, $\delta^{15}N$, $\delta^{2}H$) feather isoscape to assign Afrotropical migrant birds to origins. *Ecosphere.* **3,** 44. doi: 10.1890/ES12-00018.1 (2012).
4. Kosztra, B., Büttner, G., Hazeu, G., & Arnold, S. Updated CLC illustrated nomenclature guidelines. European Environment Agency: Copenhagen, Denmark (2017).
